# Plumbagin sensitizes breast cancer cells to tamoxifen-induced cell death through GRP78 inhibition and Bik upregulation

**DOI:** 10.1038/srep43781

**Published:** 2017-03-13

**Authors:** Anna Kawiak, Anna Domachowska, Anna Jaworska, Ewa Lojkowska

**Affiliations:** 1 Department of Biotechnology, Intercollegiate Faculty of Biotechnology, University of Gdansk and Medical University of Gdansk, Abrahama 58, Gdansk, 80-307, Poland; 2 Department of Human Physiology, Faculty of Health Sciences with Subfaculty of Nursing, Medical University of Gdansk, Tuwima 15, Gdansk, 80-210, Poland

## Abstract

The glucose regulated protein 78 (GRP78) is a major chaperone of the endoplasmic reticulum, and a prosurvival component of the unfolded protein response. GRP78 is upregulated in many types of cancers, including breast cancer. Research has suggested that GRP78 overexpression confers chemoresistance to anti-estrogen agents through a mechanism involving the inhibition of a pro-apoptotic BH3-only protein, Bik. In the present research the role of plumbagin, a naturally occurring naphthoquinone, in GRP78-associated cell death inhibition was examined. The results demonstrated that plumbagin inhibits GRP78 activity and GRP78 inhibition contributes to plumbagin-mediated cell death induction. Furthermore, Bik upregulation was associated with plumbagin-induced cell death and an increase in plumbagin-mediated Bik induction was observed upon GRP78 downregulation. Plumbagin sensitized estrogen-positive breast cancer cells to tamoxifen and the association of GRP78 inhibition and Bik upregulation in plumbagin-mediated cell sensitization was shown. Collectively, the results of this research suggest that plumbagin inhibits the antiapoptotic activity of GRP78 leading to Bik upregulation and apoptosis induction, which contributes to the sensitization of breast cancer cells to tamoxifen.

Breast cancer is one of the most common cancers diagnosed in women. Approximately 70% of breast cancer occurrences express the estrogen receptor-α^1^. Therapies aimed at targeting the estrogen receptor include selective estrogen receptor modulators (tamoxifen)^1^, estrogen antogonists (fulvestrant)^2^ or aromatase inhibitors that block the production of 17β-estradiol (anastozole)^3^. Despite the advantages of these therapies, acquired or de novo developed resistance remains a significant impediment to successful treatment of estrogen-positive cancers. The upregulation of proteins involved in stress-regulatory pathways has been associated with the development of resistance^4^. One such protein is the glucose-regulated protein GRP78 (BiP), a member of the Hsp70 family of chaperones that localizes mainly in the ER. GRP78 is a prosurvival element of the unfolded protein response, an ER related stress response^5^,^6^. The level of GRP78 has been shown to be elevated in various cancer cell lines as well as in solid tumors and biopsy samples^7^,^8^,^9^. Moreover, the overexpression of GRP78 in breast cancer patients has been correlated with resistance to chemotherapy^10^. Recent reports show that GRP78 upregulation confers resistance to antiestrogen therapy through the regulation of a BH3-only protein, Bik. Bik is a proapoptotic protein which has been shown to play a critical role in antiestrogen-mediated cell death. The upregulation of GRP78 suppresses Bik activity thus inhibiting apoptosis induction in estrogen-positive breast cancer cells^11^,^12^. These findings indicate that downregulation of GRP78 or inhibition of GRP78 activity could increase the efficacy of anti-estrogen therapy and decrease antiestrogen-mediated resistance.

Natural products are a source of highly diverse bioactive compounds which include important pharmacological leads^13^. Plumbagin (5-hydroxy-2-methyl-1,4-naphthoquinone) is a naturally occurring naphthoquinone present in plants of the genus *Drosera* and *Plumbago*^14^,^15^. Quinones are one of the largest groups of compounds used as anticancer agents^16^ and among them naphthoquinones have been shown to possess potential anticancer activity^17^,^18^,^19^. One of the most extensively researched naphthoquinones, namely plumbagin, has demonstrated high antiproliferative activity towards various cancer cell lines^20^,^21^,^22^ and its anti-cancer properties have also been shown in studies *in vivo*^23^,^24^,^25^. Plumbagin has been shown to induce apoptosis^26^,^27^ and inhibit the migration and invasion of breast cancer cells^28^,^29^,^30^,^31^,^32^. Moreover, i*n vivo* studies have shown that plumbagin significantly inhibits the growth of breast tumor xenografts in mice without toxic side-effects^24^. Previous research has shown the ability of plumbagin to induce apoptosis in estrogen-positive MCF-7 breast cancer cells^33^. Plumbagin-induced cell death was associated with the down-regulation of Bcl-2 expression^33^,^34^. Plumbagin was shown to inhibit ER-α signaling in ovarian cancer cells^35^. Furthermore, recent studies showed that plumbagin inhibits the growth of endocrine-resistant breast cancer cells and increases the sensitivity of these cells to tamoxifen-induced cell death^36^. These findings prompted us to further determine the mechanism of plumbagin-mediated sensitization of breast cancer cells to tamoxifen. The effects of plumbagin on GRP78 inhibition and its involvement in plumbagin-mediated cell death induction were examined. Furthermore, the association between GRP78 inhibition in plumbagin-mediated sensitization of breast cancer cells to tamoxifen was examined.

## Results

### Plumbagin induces apoptosis in estrogen-positive breast cancer cells

**Figure 1:**
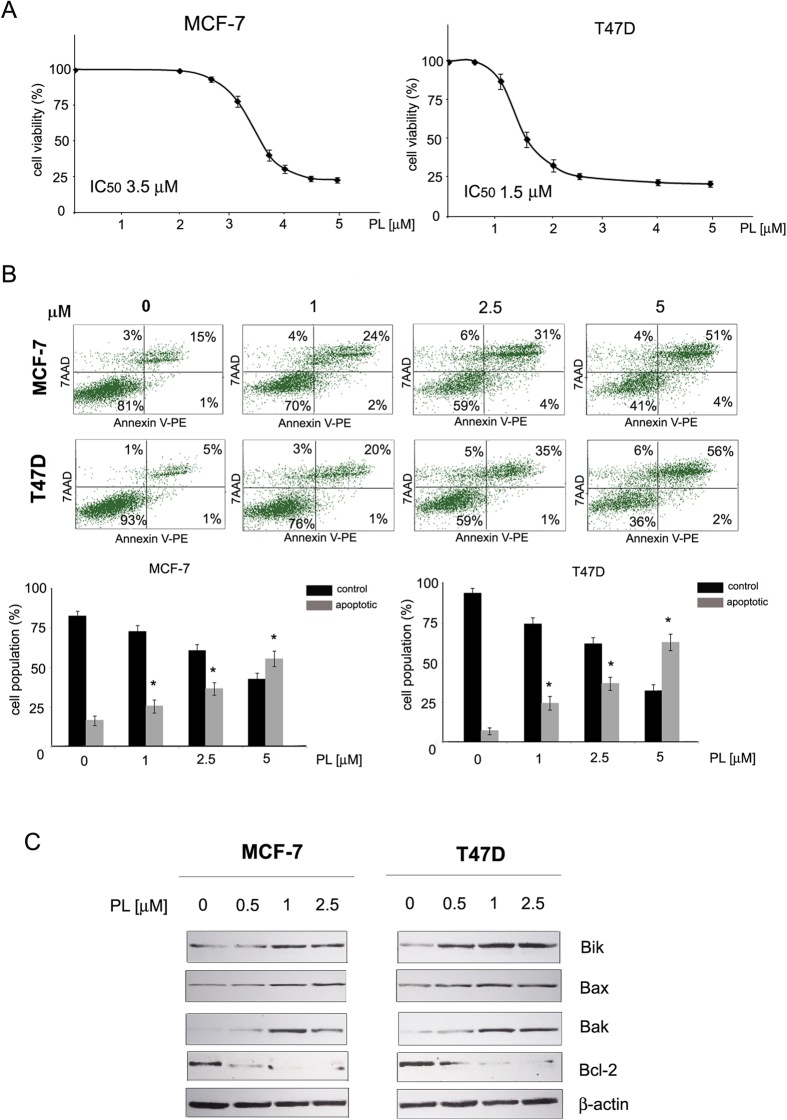
Cytotoxic and apoptosis-inducing activity of plumbagin towards ER-positive breast cancer cells. (**A**) Cytotoxic activity of plumbagin. MCF-7 and T47D cells were treated with plumbagin (0–5 μM) for 24 h with plumbagin and cell survival was assessed with the MTT assay. (**B**) Apoptotic changes in plasma membrane induced by plumbagin. Cells were treated with plumbagin (0–5 μM) for 24 h, stained with Annexin V-PE/7-AAD, and analyzed by flow cytometry. Values represent mean ± SE of three independent experiments. *p* < 0.05 (*) indicates differences between control and plumbagin-treated cells. (**C**) Effects of plumbagin on the expression levels of Bik, Bax, Bak and Bcl-2. Cells were treated with plumbagin (0–2.5 μM) for 24 h and protein levels were determined with Western blot analysis.

The effects of plumbagin on the viability of estrogen-positive breast cancer cells were determined with the MTT assay. MCF-7 and T47D cells were incubated with plumbagin in the concentration range of 0–5 μM for 24 h. The results of the MTT assay showed a dose-dependent decrease in cell viability induced by plumbagin, and the obtained IC_50_ values for MCF-7 and T47D cells were of 3.5 μM and 1.5 μM, respectively (Fig. 1A). The effects of plumbagin on apoptosis induction in estrogen-positive breast cancer cells were analyzed by determining phosphatidylserine externalization in plumbagin-treated cells. Flow cytometry analysis with Annexin V staining revealed a significant dose-dependent increase in the percentage of apoptotic cells. In accordance with the results of cytotoxic activity analysis, T47D cells showed higher sensitivity towards plumbagin and the percentage of apoptotic cells increased by around 15% and 50% at the concentration of 1 and 5 μM, respectively. In MCF-7 cells, a 10% and 40% increase in the apoptotic population of cells was induced with plumbagin concentrations of 1 and 5 μM, respectively (Fig. 1B).

Since our previous research revealed that plumbagin induces apoptosis in HER2-overexpressing breast cancer cells through the intrinsic pathway^26^, the involvement of Bcl-2 family proteins in plumbagin-induced apoptosis was examined in estrogen-positive cells. After a 24-h treatment of MCF7 and T47D cells with plumbagin, Western blot analysis of pro-apoptotic proteins (Bik, Bax, Bak) and anti-apoptotic Bcl-2 levels was carried out. The results showed that plumbagin induced the levels of pro-apoptotic Bik, Bax and Bak in MCF-7 and T47D cells. At the lowest concentration tested, namely 0.5 μM, a significant increase in the levels of pro-apoptotic proteins was observed. Conversely, the levels of anti-apoptotic protein Bcl-2 decreased upon plumbagin treatment (Fig. 1C).

### Plumbagin induces apoptosis through GRP78 inhibition

**Figure 2:**
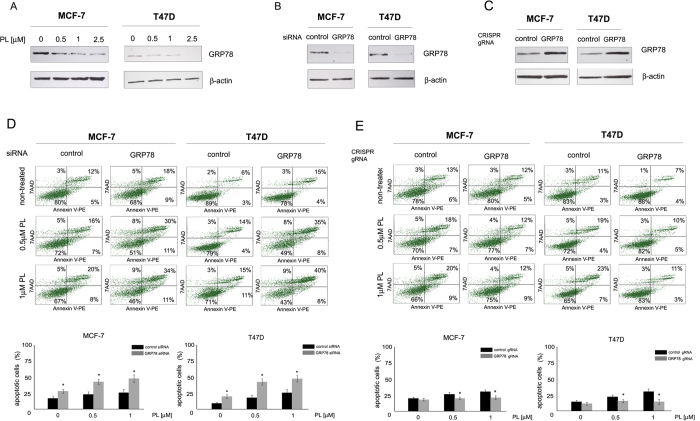
The role of GRP78 in plumbagin-mediated apoptosis induction in estrogen-positive breast cancer cells. (**A**) Effects of plumbagin on the expression levels of GRP78. MCF-7 and T47D cells were treated with plumbagin (0–2.5 μM) for 24 h and protein levels were determined with Western blot analysis. (**B**) Silencing of GRP78 in MCF-7 and T47D cells. Cells were transiently transfected with GRP78 siRNA and after 24 h GRP78 levels were determined with Western blot analysis. (**C**) Overexpression of GRP78 was performed by transiently transfecting cells with a GRP78 CRISPR/dCas9 activation plasmid. 24 h after transfection, GRP78 levels were determined with Western blot analysis. (**D**) Influence of GRP78 silencing and (**E**) GRP78 upregulation on the induction of apoptosis by plumbagin. 24 h-post transfection cells were treated with plumbagin (0.5 and 1 μM) for 24 h after which cells were stained with Annexin V-PE/7-AAD, and analyzed by flow cytometry. Values represent mean ± SE of three independent experiments. *p* < 0.05 (*) indicates differences between control and GRP78-downregulated and upregulated cells treated with plumbagin.

GRP78 plays a role in the regulation of apoptosis through inhibiting the activity of a BH3-only protein – Bik. Since plumbagin was shown to induce Bik expression, the influence of plumbagin on GRP78 levels in estrogen-positive breast cancer cells was determined. MCF-7 and T47D cells were treated with plumbagin in the concentration range of 0–2.5 μM and Western blot analysis revealed a significant inhibition in GRP78 expression in plumbagin-treated cells. At the lowest concentration tested, 0.5 μM, a significant decrease in GRP78 expression was observed and GRP78 levels decreased with increasing concentrations of plumbagin (Fig. 2A).

The involvement of GRP78 inhibition in plumbagin-mediated apoptosis induction in estrogen-positive breast cancer cells was examined by transiently silencing or upregulating GRP78 expression and determining the percentage of apoptotic cells upon plumbagin treatment. GRP78 downregulation was performed by transiently transfecting cells with GRP78 siRNA, whereas a GRP78 CRISPR/dCas9 activation plasmid was used for GRP78 upregulation. Western blot analysis was performed in order to verify GRP78 levels in transfected cells. The results, presented in Fig. 2B, show that GRP78 expression was significantly reduced in siRNA transfected MCF-7 and T47D cells, whereas a significant upregulation of GRP78 expression was determined in cells transfected with a GRP78 CRISPR/dCas9 activation plasmid (Fig. 2C). To determine the involvement of GRP78 in plumbagin-mediated cell death induction in breast cancer cells, 24 h after transfection, cells were treated with plumbagin and apoptosis induction was assessed with Annexin V-PE staining. A significant increase in the percentage of apoptotic cells was observed in cells with reduced GRP78 expression in comparison with control cells (Fig. 2D). In cells overexpressing GRP78, a decrease in the percentage of apoptotic cells was observed upon plumbagin treatment (Fig. 2E). These results indicate the involvement of GRP78 in plumbagin-mediated apoptosis induction in estrogen-positive breast cancer cells.

### Plumbagin sensitizes estrogen-positive breast cancer cells to tamoxifen

**Figure 3:**
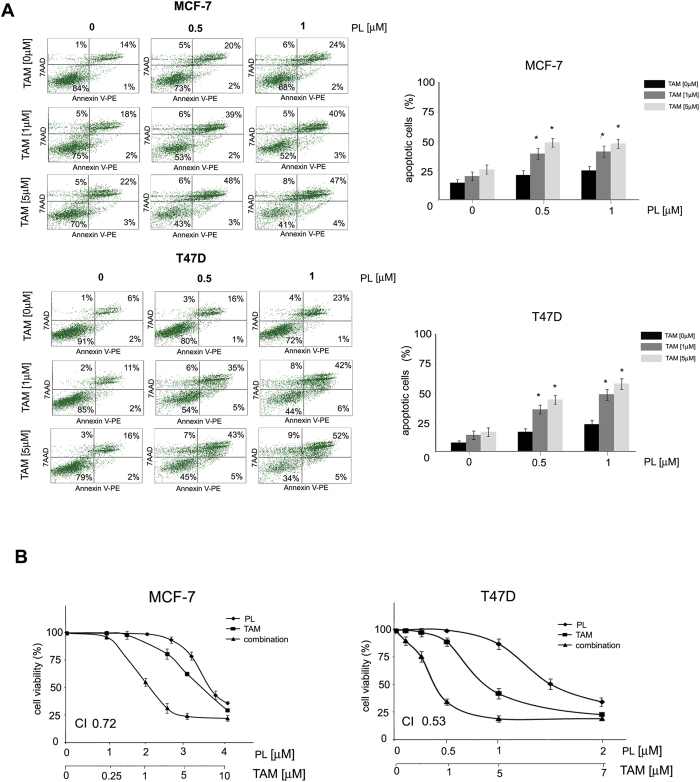
Plumbagin sensitizes breast cancer cells to tamoxifen. (**A**) Influence of plumbagin on tamoxifen-induced apoptosis. Cells were incubated with a combination of plumbagin (0.5 or 1 μM) and tamoxifen (1 and 5 μM) for 24 h after which cells were stained with Annexin V-PE/7-AAD, and analyzed by flow cytometry. Values represent mean ± SE of three independent experiments. *p* < 0.05 (*) indicates differences between single agent-treated and combination-treated cells. (**B**) Synergistic effect of plumbagin and tamoxifen on estrogen-positive breast cancer cell viability. MCF-7 and T47D cells were exposed to combinations of plumbagin and tamoxifen. Cell viability was assessed 24 h after exposure with the MTT assay.

In view of the fact that plumbagin inhibits GRP78 activity and that GRP78 has been implicated in anti-estrogen induced resistance, the effects of plumbagin on tamoxifen-induced cell death were examined. MCF-7 and T47D cells were treated with plumbagin at the concentrations of 0.5 μM or 1 μM in the presence of tamoxifen (1 μM or 5 μM). Cells were treated for 24 h and cell death was analyzed with Annexin V staining. Plumbagin was shown to increase the sensitivity of estrogen-positive breast cancer cells to tamoxifen. Tamoxifen increased the percentage of apoptotic cells by 5% to 10% in MCF-7 nad T47D cells, respectively, whereas the addition of plumbagin increased apoptosis induction by 10–15% in MCF-7 cells, and 15–20% in T47D cells (Fig. 3A).

To further determine the possible synergistic activity between plumbagin and tamoxifen, the median-effect method^37^ was used to analyze the combined effect of plumbagin and tamoxifen. MCF-7 and T47D cells were treated with five dose combinations of plumbagin (μM)/tamoxifen (μM) at a fixed ratio, including 1/0.25, 2/1, 2.5/3, 3/5, 4/10 for MCF-7 cells and 0.1/0.25, 0.25/0.5, 0.5/1, 1/5, 2/7 for T47D cells, versus varying concentrations of plumbagin (0–4 μM) and tamoxifen (0–10 μM). The dose-responsive curves are shown in Fig. 3B. The CI (combination index) values obtained for the examined cell lines were below 1 indicating synergistic activity between plumbagin and tamoxifen in estrogen-positive breast cancer cells. The CI values obtained for the combination of plumbagin and tamoxifen that caused a 50% decrease in cell viability were 0.72 and 0.53 for MCF-7 and T47D cells, respectively.

### Plumbagin sensitizes breast cancer cells to tamoxifen through GRP78 inhibition and Bik upregulation

**Figure 4:**
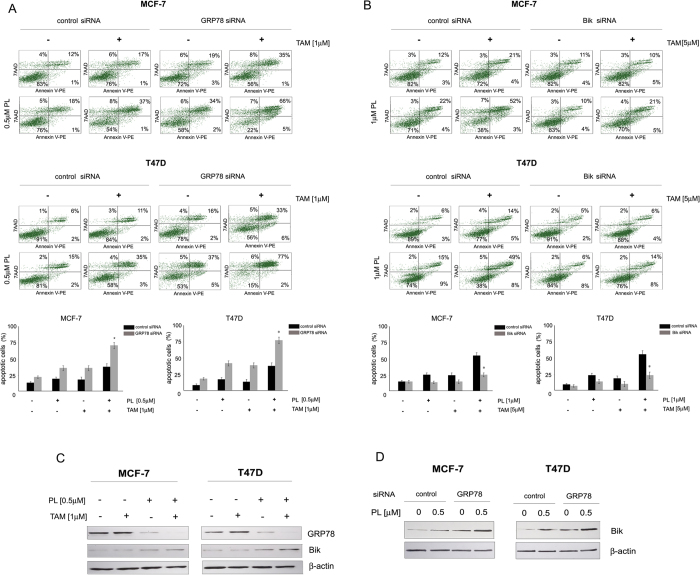
The involvement of GRP78 and Bik in the induction of apoptosis by combination treatment of breast cancer cells with plumbagin and tamoxifen. MCF-7 and T47D cells were transiently transfected with (**A**) GRP78 siRNA or (**B**) Bik siRNA. 24 h-post transfection cells were treated for an additional 24 h with plumbagin and tamoxifen. Cells were stained with Annexin V-PE/7-AAD and analyzed by flow cytometry. Values represent mean ± SE of three independent experiments. *p* < 0.05 (*) indicates differences between combination-treated control and GRP78 or Bik-downregulated cells. (**C**) Influence of combination treatment with plumbagin and tamoxifen on expression levels of GRP78 and Bik. Cells were treated for 24 h with plumbagin (0.5 μM) and tamoxifen (1 μM) and GRP78 or Bik levels were determined with Western blot analysis. (**D**) Influence of GRP78 silencing on plumbagin-mediated Bik induction. 24 h-post transfection cells were treated with plumbagin (0.5 μM) for 24 h and the expression levels of Bik were determined with Western blot analysis.

In order to determine whether the sensitizing effect of plumbagin to tamoxifen-induced apoptosis is associated with the ability of plumbagin to inhibit GRP78 and upregulate Bik activity, apoptosis-inducing effects of plumbagin and tamoxifen combination treatment were evaluated in cells with reduced GRP78 and Bik expression. MCF-7 and T47D cells were transiently transfected with GRP78 siRNA or Bik siRNA. Cells were treated with a combination of plumbagin (0.5 μM or 1 μM) and tamoxifen (1 μM or 5 μM). Following a 24 h-treatment cell death was analyzed with Annexin V staining. The results of cytometric analysis showed a significant increase in the percentage of apoptosis induced in cells with silenced GRP78 in comparison with cells transfected with scrambled siRNA. In cells with reduced GRP78 expression combination treatment with plumbagin and tamoxifen increased the population of apoptotic cells by around 10% in MCF-7 and 15% in T47D cells (Fig. 4A). In the case of Bik downregulation, a significant decrease in the percentage of apoptotic cell populations was observed in cells with decreased Bik expression treated with a combination of plumbagin and tamoxifen, in comparison with control cells (Fig. 4B). To further confirm the involvement of GRP78 inhibition in the synergistic activity of plumbagin and tamoxifen, the influence of combined treatment with these compounds on GRP78 and Bik expression levels was determined. The results showed that the treatment of MCF-7 and T47D cells with both plumbagin and tamoxifen significantly reduced expression levels of GRP78 and increased the levels of Bik, in comparison with single agent treatment (Fig. 4C). These results suggest the involvement of GRP78 inhibition and Bik upregulation by plumbagin in its sensitizing effects to tamoxifen in estrogen-positive breast cancer cells.

The influence of GRP78 in the regulation of plumbagin-induced Bik upregulation was further examined. Cells transiently transfected with GRP78 siRNA or control siRNA were treated with plumbagin (0.5 μM) and after a 24-h incubation, Western blot analysis was performed in order to determine Bik levels. GRP78 silencing increased Bik levels in MCF-7 and T47D cells. Furthermore, the treatment of cells with plumbagin induced Bik expression in GRP78-silenced cells in comparison with control cells transfected with control siRNA (Fig. 4D). These results point to the involvement of GRP78 regulation in Bik-mediated plumbagin induction of cell death.

## Discussion

The endoplasmic reticulum (ER) has emerged as a novel regulatory site for antiestrogen-induced apoptosis as well as estrogen-mediated resistance^38^. A key mediator involved in endoplasmic reticulum-regulated apoptosis is the Bcl-2-interacting killer (Bik), a member of the BH3-only proteins. Bik is a pro-apoptotic protein mainly localized to the outer ER membrane and induces apoptosis through the mitochondrial pathway. An increase in the levels of Bik enable the formation of Bik/Bcl-2 complexes at the ER. Binding of Bcl-2 and its inactivation promotes Ca^2+^ release from the ER, initiating the apoptotic process^12^. Research has shown that Bik plays a central role in antiestrogen induced apoptosis. In a cell line dependent on estrogen for growth, antiestrogen treatment strongly induced Bik expression without affecting the expression of other Bcl-2 family proteins^39^. Bik is regulated at the ER through interactions with the glucose-regulated protein GRP78. GRP78 is a key prosurvival component of the unfolded protein response (UPR), it participates in proper protein folding and targets misfolded proteins for degradation. GRP78 is a endoplasmic reticulum stress regulator, controlling Ca^2+^ binding and activating transmembrane endoplasmic reticulum stress inducers^40^,^41^. GRP78 is upregulated in many types of tumor cells including breast cancer cells^10^,^42^. High levels of GRP78 in tumors have been correlated with the altered metabolism of cancer cells including higher glucose utilization and increased glycolytic activity. Glucose and oxygen deprivation leads to the accumulation of underglycosylated and misfolded proteins in the ER activating the UPR for cell survival^43^.

The involvement of GRP78 in resistance to estrogen-induced apoptosis in breast cancer cells has been associated with its anti-apoptotic properties and ability to complex with Bik. GRP78, under stress conditions such as long-term estrogen deprivation, sequesters Bik through complex formation. Sequestered Bik has reduced ability to bind to Bcl-2, thus increasing Bcl-2 levels which suppresses the release of Ca^2+^ from the ER and inhibits apoptosis induction^11^,^12^. Research has shown that the overexpression of GRP78 suppresses Bik-induced apoptosis, whereas GRP78 knockdown restores sensitivity of cells to apoptosis induction^11^. In accordance, our results showed that GRP78 silencing upregulates Bik expression in estrogen-positive breast cancer cells. Furthermore, the inhibition of GRP78 by plumbagin treatment induced apoptosis in breast cancer cells. GRP78 silencing increased the sensitivity of cells to plumbagin-induced cell death. Previous research has shown the ability of plumbagin to induce cell death in estrogen-positive cells and the mitochondria-mediated pathway was implicated in cell death induction. Ahmad *et al*.^33^ showed that plumbagin induces apoptosis in MCF-7 cells through targeting Bcl-2. Similarly, Sagar *et al*.^34^ showed the down-regulation of *BCL-2* gene expression in MCF-7 cells. In accordance, the present research shows the involvement of the intrinsic pathway in plumbagin-mediated apoptosis induction in estrogen-positive breast cancer cells. An increase in Bak and Bax and a decrease in Bcl-2 levels were observed. Importantly, the involvement of Bik upregulation in plumbagin-mediated cell death was demonstrated in the present research. Plumbagin-induced Bik upregulation was found to be controlled by GRP78 as shown by an increased induction of Bik by plumbagin in cells with GRP78 downregulation.

GRP78 is frequently overexpressed in refractory tumors and research has pointed to the activation of the pro-survival mechanism and GRP78 upregulation as being a significant contributing factor in the development of antiestrogen-induced resistance in cancer cells. Increased GRP78 levels have been reported in cell lines resistant to antiestrogen treatment and increased expression of GRP78 was also observed in antiestrogen-resistant breast cancer xenografts in comparison with xenografts sensitive to treatment. The silencing of GRP78 restored sensitivity of resistant estrogen-positive tumors and cell lines to antiestrogen drugs, such as tamoxifen^44^. Thus, these findings suggest that targeting GRP78 during antiestrogen treatment could improve the outcome of estrogen-positive breast cancer therapy. In the present research plumbagin was demonstrated to sensitize breast cancer cells to tamoxifen through a synergistic activity between both compounds. Plumbagin increased tamoxifen-mediated apoptosis induction and the involvement of GRP78 in combination treatment-induced cell death was demonstrated. The present research suggests that plumbagin could have potential in estrogen-positive breast cancer therapy due to its ability to sensitize breast cancer cells to tamoxifen through GRP78 inhibition.

In conclusion, the research presented herein shows that plumbagin induced apoptosis in estrogen-positive breast cancer cells through GRP78 inhibition. Plumbagin-mediated GRP78 inhibition upregulated Bik and induced apoptosis through the mitochondria-regulated apoptotic pathway. Furthermore, plumbagin sensitized breast cancer cells to tamoxifen-induced cell death and the involvement of GRP78 inhibition was associated with plumbagin-mediated sensitization. Thus our findings provide a rationale for further research on plumbagin as a potential agent in the treatment of estrogen-positive breast cancer and in combination treatment with antiestrogen agents.

## Methods

### Chemicals

Plumbagin was obtained at >95% purity from Sigma-Aldrich (St. Louis, MO, USA). All cell culture materials and other chemicals, if not indicated otherwise were obtained from the same company.

### Cell culture

The MCF-7 and T47D breast cancer cell lines were purchased from Cell Lines Service (Germany). Cells were cultured in RPMI medium supplemented with 10% fetal bovine serum, 2 mM glutamine, 100 units/mL penicillin and 10 mg/mL streptomycin. Cultures were maintained in a humidified atmosphere with 5% CO_2_ at 37 °C in an incubator (Heraceus, Hera cell).

### Cytotoxicity assay

The influence of plumbagin on estrogen-positive breast cancer cell viability was determined using the MTT [(3-(4,5-dimethylthiazol-2-yl)-2,5-diphenyltetrazolium bromide] assay. Cells were treated with plumbagin in the concentration range of 0–5 μM and/or with tamoxifen (0-10 μM) for 24 h. Analysis was performed according to the previously published procedure^45^.

### Annexin V-PE staining

Apoptosis induction was examined with an Annexin V-PE Apoptosis Detection Kit I (BD Biosciences, Belgium). The procedures were carried out according to the manufacturer’s instructions. Briefly, MCF-7 and T47D cells were treated with plumbagin (0–5 μM) and/or with tamoxifen (0-5 μM) for 24 h, after which cells were collected, washed with Annexin-binding buffer, and stained with Annexin V- phycoerythrin (PE) and 7-amino-actinomycin (7-AAD). Following a 30 min incubation at 15 °C in the dark, samples were analyzed by flow cytometry (BD FACSCalibur).

### Western blot analysis

MCF-7 and T47D cells were treated with plumbagin (0–2.5 μM) and/or with tamoxifen (1 μM) for 24 h and Western blot analysis was performed according to the previously published procedure. Specific primary antibodies: anti-GRP78, anti-Bik, anti-Bax, anti-Bak, anti-Bcl-2 (1:250) (Santa Cruz, Heidelberg, Germany), anti-β-actin (1:1000) (Cell Signaling, Germany), were incubated with membranes overnight at 4 °C. Membranes were further incubated at room temperature for 1 h with HRP-conjugated secondary antibodies (Cell Signaling, Germany) and proteins were detected by chemiluminescence (ChemiDoc, Bio-Rad) with a HRP substrate (Pierce).

### GRP78and Bik silencing

The expression of GRP78 or Bik was silenced in MCF-7 and T47D cells using GRP78 siRNA or Bik siRNA and control, scrambled siRNA (Santa Cruz, Germany). Cells were transiently silenced with siRNA (0.5 μg) using siRNA Transfection Reagent (Santa Cruz, Germany) according to the manufacturer’s instructions. 24 h post-transfection, GRP78 and Bik silencing was verified with Western blot analysis or cells were treated with a combination of plumbagin and tamoxifen versus single treatment with plumbagin or tamoxifen. Samples were analyzed by flow cytomtery following Annexin V-PE staining to determine apoptosis induction.

### GRP78 overexpression

The overexpression of GRP78 was carried out with a GRP78 CRISPR/dCas9 activation plasmid (Santa Cruz, Germany). Cells were transiently transfected with a GRP78 CRISPR/dCas9 activation plasmid (1 μg) or a control scrambled CRISPR activation plasmid using the UltraCruz Transfection Reagent (Santa Cruz, Germany), according to the manufacturer’s instructions. 24 h post-transfection, GRP78 overexpression was verified with Western blot analysis or cells were treated with plumbagin. Samples were analyzed by flow cytometry following Annexin V-PE staining to determine apoptosis induction.

### Synergism determination

The synergistic effect of plumbagin and tamoxifen was determined by the method of Chou and Talalay^37^. Cells were treated with a series of plumbagin and tamoxifen concentrations at fixed ratios. The drug concentrations ranged from 0.25 to 10 μM. The values of CI at different levels of growth inhibition were calculated based on the formula for mutually nonexclusive mechanism: (D_1_/Dx_1_) + (D_2_/Dx_2_) + (D_1_D_2_/Dx_l_Dx_2_), where Dx_1_ and Dx_2_ are the doses of drug 1 and drug 2 alone required to produce *x* percentage effect, and D_1_ and D_2_ are the doses of drug 1 and drug 2 in combination required to produce the same effect. The CI values less than 1, equal to 1, and greater than 1 are indicative of synergism, additive effect, and antagonism, respectively.

### Statistical analysis

Values are expressed as means ± SE of at least three independent experiments. Statistical analysis was performed using GraphPad Prism 5.0 (GraphPad software). Differences between control and single agent-treated samples were analyzed by one-way ANOVA with Tukey’s post-hoc tests. Differences between single and double agent treatments were analyzed by two-way ANOVA with Bonferoroni post-tests. A *p* value of <0.05 was considered as statistically significant in each experiment.

## Additional Information

**How to cite this article**: Kawiak, A. *et al*. Plumbagin sensitizes breast cancer cells to tamoxifen-induced cell death through GRP78 inhibition and Bik upregulation. *Sci. Rep.* **7**, 43781; doi: 10.1038/srep43781 (2017).

**Publisher's note:** Springer Nature remains neutral with regard to jurisdictional claims in published maps and institutional affiliations.
